# Comparison of Computed Tomography derived Fractional Flow Reserve to invasive Fractional Flow Reserve in Diagnosis of Functional Coronary Stenosis: A Meta-Analysis

**DOI:** 10.1038/s41598-018-29910-9

**Published:** 2018-08-01

**Authors:** Pradyumna Agasthi, Arun Kanmanthareddy, Charl Khalil, Obiora Egbuche, Vivek Yarlagadda, Rajesh Sachdeva, Reza Arsanjani

**Affiliations:** 1 Division of Cardiovascular Diseases, Mayo Clinic Arizona, Phoenix, Arizona USA; 2 Division of Cardiovascular Medicine, University of Texas Health Science Center at Houston, Houston, Texas USA; 3 Division of Cardiology, Department of Medicine, Morehouse School of Medicine, Atlanta, Georgia; 4 Department of Internal Medicine, Atlanticare Regional Medical Center, Atlantic City, New Jersey USA

## Abstract

Computed Tomography derived Fractional Flow Reserve (CTFFR) is an emerging non-invasive imaging modality to assess functional significance of coronary stenosis. We performed a meta-analysis to compare the diagnostic performance of CTFFR to invasive Fractional Flow reserve (FFR). Electronic search was performed to identify relevant articles. Pooled Estimates of sensitivity, specificity, positive likelihood ratio (LR+), negative likelihood ratio (LR−) and diagnostic odds ratio (DOR) with corresponding 95% confidence intervals (CI) were calculated at the patient level as well as the individual vessel level using hierarchical logistic regression, summary receiver operating characteristic (SROC) curve and area under the curve were estimated. Our search yielded 559 articles and of these 17 studies was included in the analysis. A total of 2,191 vessels in 1294 patients were analyzed. Pooled estimates of sensitivity, specificity, LR+, LR− and DOR with corresponding 95% CI at per-patient level were 83% (79–87), 72% (68–76), 3.0 (2.6–3.5), 0.23 (0.18–0.29) and 13 (9–18) respectively. Pooled estimates of sensitivity, specificity, LR+, LR− and DOR with corresponding 95% CI at per-vessel level were 85% (83–88), 76% (74–79), 3.6 (3.3–4.0), 0.19 (0.16–0.22) and 19 (15–24). The area under the SROC curve was 0.89 for both per patient level and at the per vessel level. In our meta-analysis, CTFFR demonstrated good diagnostic performance in identifying functionally significant coronary artery stenosis compared to the FFR.

## Introduction

Coronary computed tomography angiography (CCTA) is a non-invasive imaging test to evaluate the burden of coronary artery disease (CAD) and has a high sensitivity and diagnostic accuracy in excluding obstructive CAD in low to intermediate risk patients^1,2^. However, CCTA has low specificity and has low positive predictive value for determining the functional significance of the lesions identified and remains a fundamental weakness of this test^3^. Less than half of the obstructive lesions identified by CCTA were associated with functional ischemia upon evaluation by invasive coronary angiography (ICA)^4–6^. This therefore may lead to unnecessary invasive coronary angiography (ICA) and/or additional unnecessary testing^7,8^.

Invasive assessment of the functional significance of the coronary stenosis is evaluated using fractional flow reserve (FFR). Several randomized control trials have demonstrated the clinical utility and long-term mortality benefit using FFR-guided revascularization, which has been adapted widely in clinical practice^9–13^. more recent studies have demonstrated that FFR could be estimated from the CCTA study using novel iteration techniques and can determine the functional significance of coronary stenotic lesions^14^. The CT guided FFR (CTFFR) approach utilizes application of computational fluid dynamics to CCTA images to determine the functional severity of the lesions^15,16^. CTFFR estimates virtual hyperemia across a lesion by using computational flow modeling without the need for vasodilator agents^17^. Several studies have reported feasibility and diagnostic performance of CTFFR^14,18–33^. We performed this meta-analysis to compare the diagnostic performance of CTFFR in comparison to the gold standard FFR.

## Results

### Eligible studies

**Figure 1:**
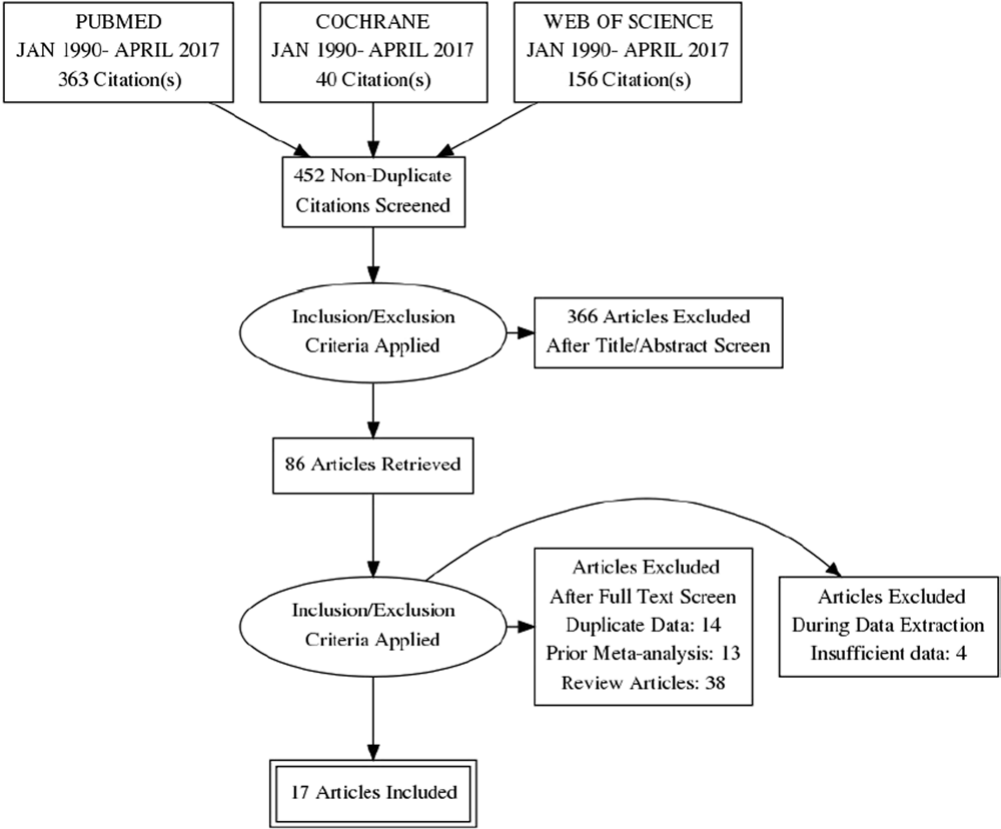
PRISMA Flow Chart.

Our search yielded 559 articles with relevant publications. Most publications were not relevant for the meta-analysis given the use of a broad search strategy. After exclusion of duplicates, 452 articles remained and further 366 articles were excluded based on the review of title and the abstract. After final review and application of inclusion and exclusion criteria, seventeen studies were included in the analysis. Figure 1 details our exclusive search results and the exclusion process.

Of the seventeen studies that were included, four were multicenter prospective studies^14,18–20^, six were single center prospective studies^24,26–28,30,33^ and seven were single-center retrospective studies^21–23,25,29,31,32^. Eight studies reported diagnostic accuracy of CTFFR on a per-patient basis and 17 studies reported on an individual vessel level basis. Study and baseline characteristics are detailed in Tables 1 and S1. Individual study estimates of diagnostic accuracy of CTFFR to identify ischemia-causing lesions are detailed in Supplementary Table S2 for per-patient level, while Supplementary Table S3 details per-vessel diagnostic accuracy results. The overall quality of included studies per quality assessment of diagnostic accuracy studies tool (QUADAS-2) was good (Supplementary Figs S1–S3).

**Table 1:** Study Characteristics.

Author		Year	Number of Patients	Number of lesions/vessels	Age (Yrs.) (Mean ± SD)		Male %	Cut off Value	
			Total	Total	Mean	SD		FFR	FFR(CT)
Koo^14^	Multicenter prospective	2011	103	159	62.7	9	72	<0.8	<0.8
Min^18^	Multicenter prospective	2012	252	407	62.9	9	71	<0.8	<0.8
Norgaard^19^	Multicenter prospective	2014	254	484	64	10	64	<0.8	<0.8
Kim^20^	Multicenter retrospective	2014	44	48	65	9	80	<0.8	<0.8
Renker^21^	Single-center retrospective	2014	53	67	61.2	12	64	<0.8	<0.8
Coenen^22^	Single-center retrospective	2015	106	189	61.4	9	77	<0.8	<0.8
De Geer^23^	Single-center retrospective	2015	21	23	60	40–74	52.4	<0.8	<0.8
Kruk^24^	Single-center Prospective	2016	90	96	63.4	8	32	<0.8	<0.8
Zhang^25^	Single-center Retrospective	2016	21	32	52	10	76	<0.8	<0.8
Gaur^26^	Single-center Prospective	2017	60	124	61	10	83	<0.8	<0.8
Kawaji^27^	Single-center Prospective	2017	43	70	70.8	8	65	<0.8	<0.8
Ko^28^	Single-center Prospective	2017	30	56	60	9	70	<0.8	<0.8
Kurata^29^	Single-center Retrospective	2017	21	29	69.6	9	76	<0.8	<0.8
Osawa^30^	Single-center Prospective	2017	20	26	73	8	80	<0.8	<0.8
Packard^31^	Single-center Retrospective	2017	75	207	66	10	75	<0.8	<0.8
Shi^32^	Single-center Retrospective	2017	29	36	68.1	8	55.2	<0.8	<0.8
Yang^33^	Single-center Prospective	2017	72	138	62.7	9	89	<0.8	<0.8

### Data Synthesis

Our study included a total of 17 studies with 1,294 patients. Among the 1,294 patients, a total of 2,191 individual vessels were assessed using FFR and CTFFR. The FFR was chosen as the gold standard and the results of CTFFR as expressed in reference to the FFR.

#### Patient Level Analysis

**Figure 2:**
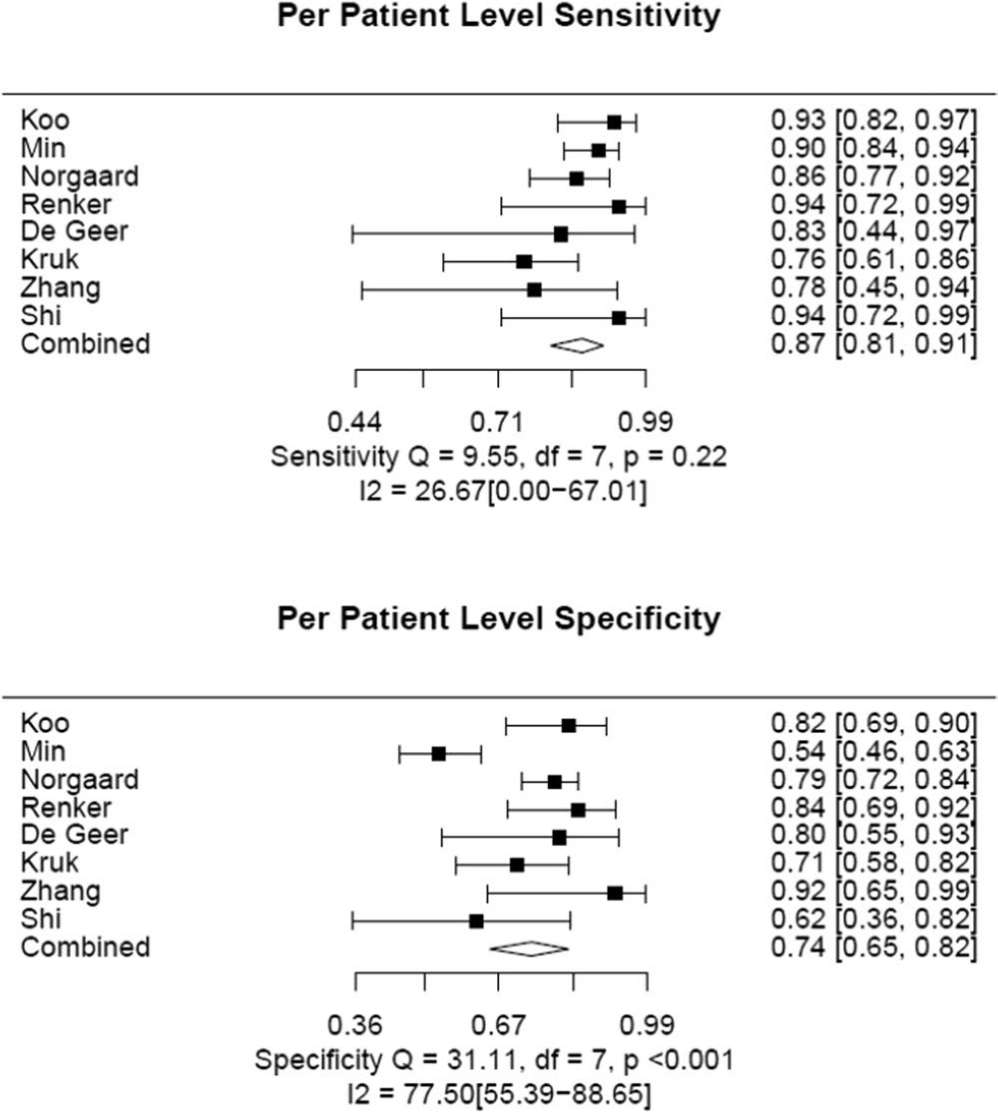
Patient Level Analyses of Sensitivity and Specificity of CTFFR vs FFR.

**Figure 3:**
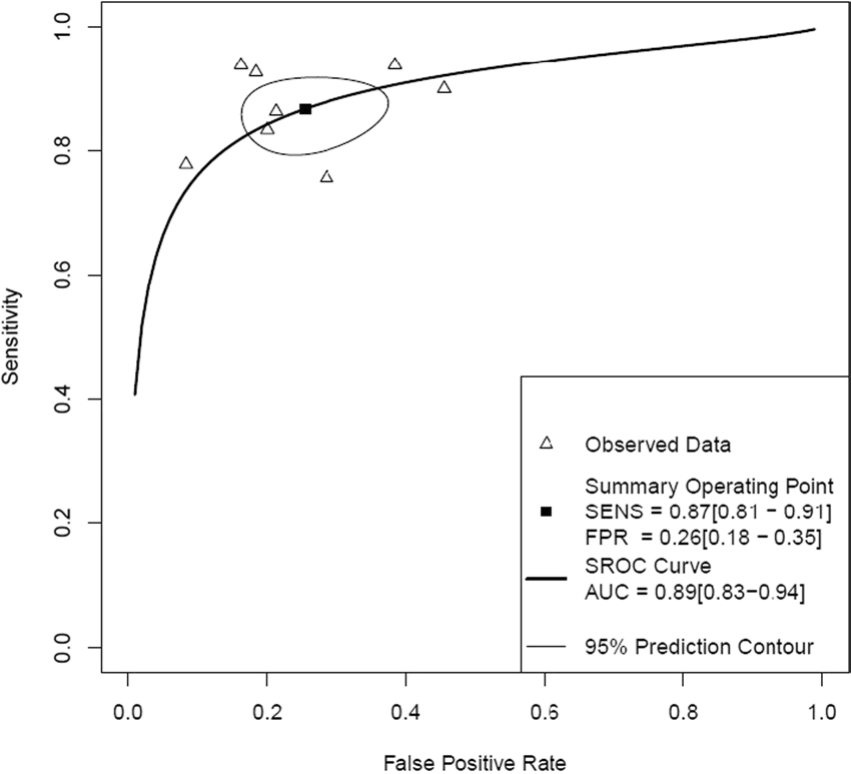
Patient Level Hierarchical Summary Receiver Operating Curve of CTFFR vs FFR.

The pooled estimates of sensitivity and specificity for CTFFR were 83% (95% CI: 79–87) and 72% (95% CI: 68–76), respectively (Fig. 2 and Table 2). The positive likelihood ratio (LR+), negative likelihood ratio (LR−) and diagnostic odds ratio (DOR) for CTFFR with respect to FFR were 3.0 (95% CI: 2.6–3.5), 0.23 (95% CI: 0.18–0.29) and 13% (95% CI: 9–18), respectively (Table 2). The area under the curve of the summary receiver operating characteristic curve (SROC) for CTFFR was 0.89 (95% CI 0.83–0.94) demonstrating good fit (Fig. 3).

**Table 2:** Pooled sensitivity, specificity, LR+, LR− and DOR of CTFFR.

Analysis Level	No. of Studies	Combined Data	Sensitivity	Specificity	LR+	LR−	DOR
Per-patient	8	1294	83% (79–87)^*^	72% (68–76)	3.0 (2.6–3.5)	0.23 (0.18–0.29)	13 (9–18)
Per-vessel	17	2191	85% (83–88)	76% (74–79)	3.6 (3.3–4.0)	0.19 (0.16–0.22)	19 (15–24)

LR+ = positive likelihood ratio; LR− = negative likelihood ratio; DOR = diagnostic odds ratio; *Numbers in parentheses are 95% confidence intervals (CIs).

#### Individual Coronary Vessel Level Analysis

**Figure 4:**
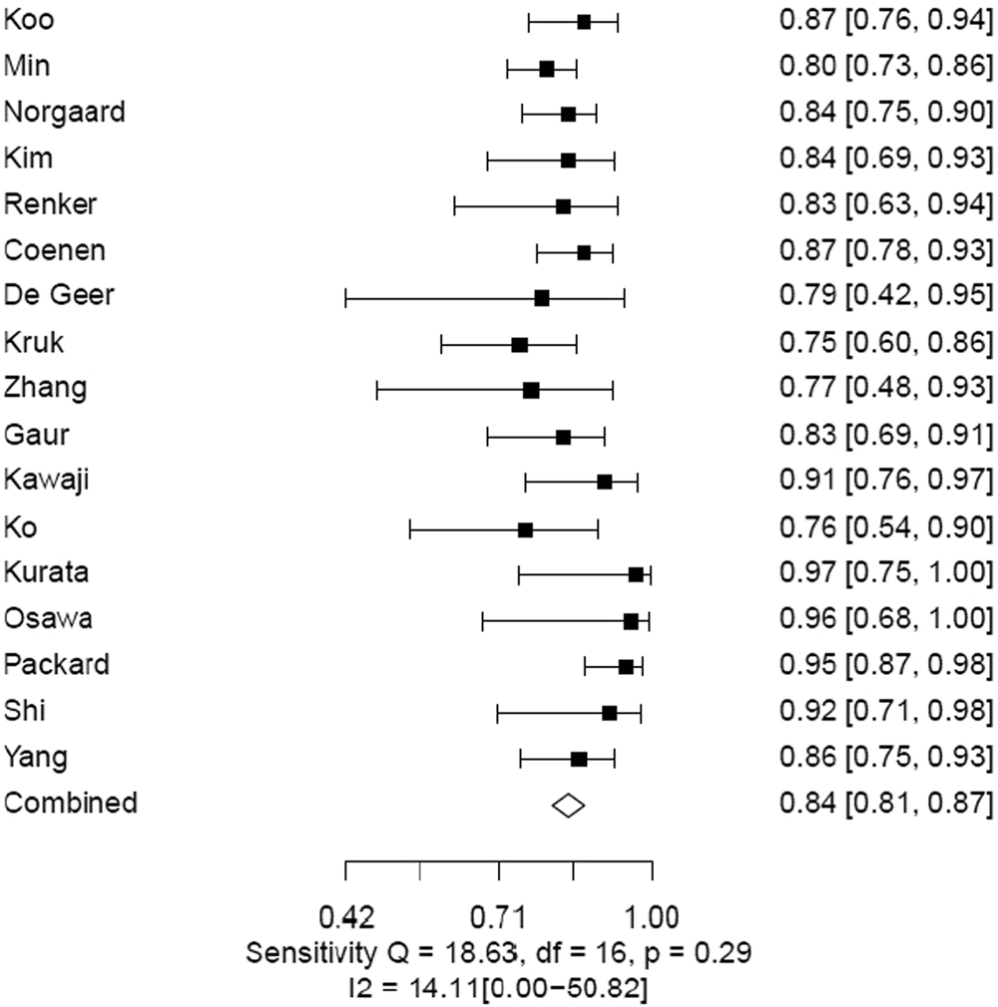
Individual Vessel Level Analyses of Sensitivity of CTFFR vs FFR.

**Figure 5:**
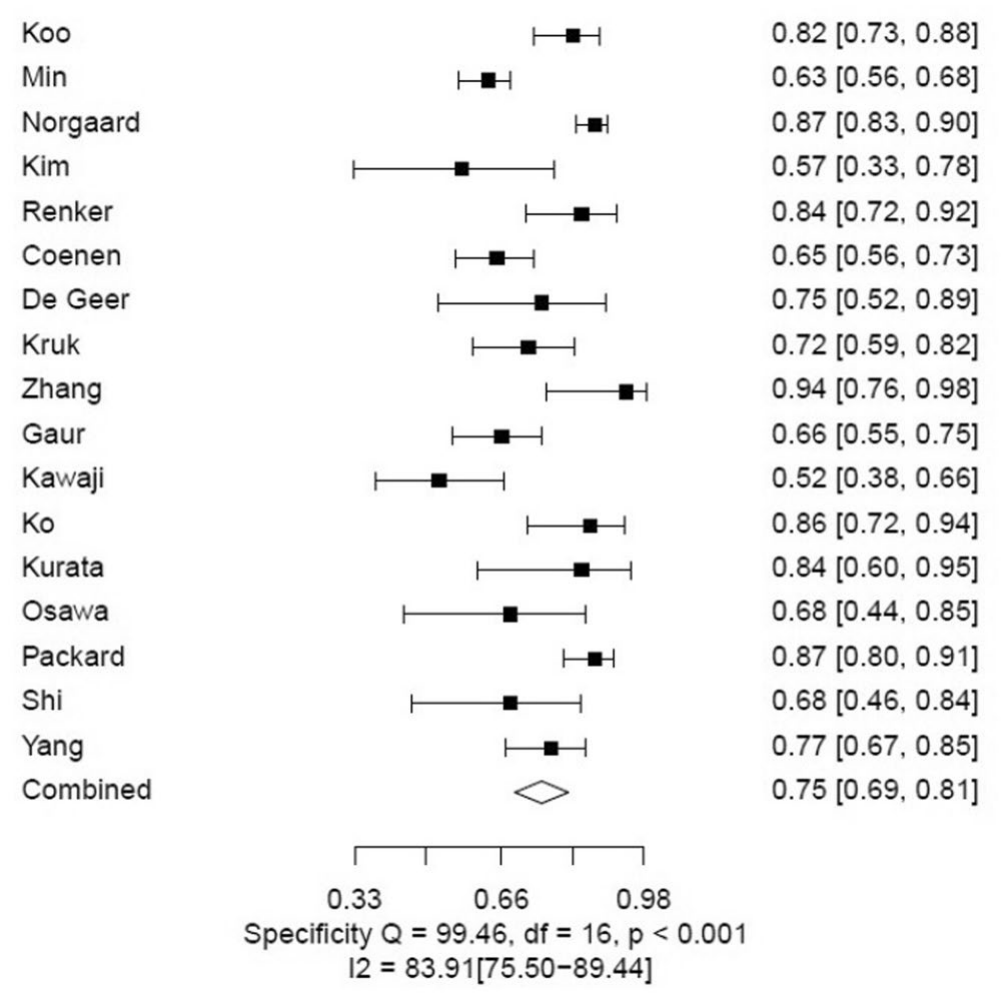
Individual Vessel Level Analyses of Specificity of CTFFR vs FFR.

**Figure 6:**
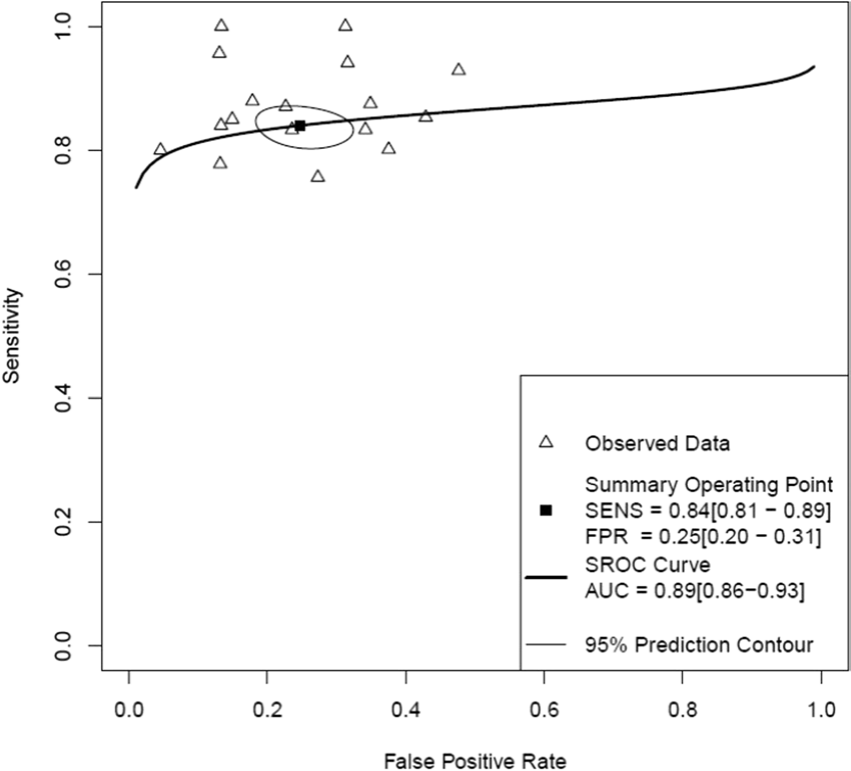
Individual Vessel level Hierarchical Summary Receiver Operating Curve of CTFFR vs FFR.

The pooled estimates of sensitivity and specificity for CTFFR with respect to FFR on an individual vessel level analyses were 85% (95% CI 83–88) and 76% (95% CI 74–79), respectively (Figs 4, 5 and Table 2). The LR+, LR− and DOR were 3.6 (95% CI 3.3–4.0), 0.19 (95% CI 0.16–0.22) and 19 (95% CI 15–24) (Supplementary Table S3). The area under the curve at the individual vessel level analysis of the SROC for the CTFFR was 0.89 (95% 0.86–0.93) suggesting good diagnostic accuracy compared to FFR (Fig. 6).

Further, posterior estimation using Bayes Nomogram was performed. At the patient level analysis using a pre-test probability of disease of 25%, a positive result on CTFFR would increase the likelihood of disease to 53%, whereas a negative test would decrease the likelihood of disease to 5% (Supplementary Fig. S4). When individual vessel level analysis was repeated, the results were similar (Supplementary Fig. S5). This therefore suggests a very high negative predictive value for exclusion of hemodynamically significant stenosis with CTFFR.

### Publication bias

Assessment for publication bias was performed using Deek’s funnel plot asymmetry test. There was no evidence of publication bias at both per patient (p = 0.48) and per vessel level (p = 0.86) analyses. However, given the low sensitivity of the Deek’s funnel plot as well as the small number of studies included in this meta-analysis, publication bias cannot be absolutely eliminated.

### Meta-regression

Multivariate meta-regression analysis was performed at per-patient level and per-vessel level for the covariates including study design, sample size (less or more than 100), year of publication, region of study, quality of study, method of CTFFR appraisal, prevalence of CAD, proportion of hypertensive patients, proportion of diabetic patients, proportion of smokers and proportion of patients with dyslipidemia to identify potential sources of heterogeneity (Supplementary Table S4). These exploratory variables were not found to have a significant impact on the heterogeneity of the results at both patient level as well as individual vessel level (Supplementary Table S5).

## Discussion

The results of our meta-analysis suggest that CTFFR has good diagnostic accuracy in detecting functionally significant coronary stenosis when compared to the FFR. These findings remained consistent at both per-patient and per-vessel level analysis.

CCTA is useful for noninvasive assessment of CAD in stable ischemic heart disease, as well as in patients with acute onset chest pain and acute coronary syndromes^34–36^. Studies have demonstrated its clinical utility and prognostic value in patients with suspected CAD with a very high negative predictive value^37^. However, CCTA overestimates the severity of stenotic lesions with only a minority of identified lesions being functionally ischemic therefore leading to increased utilization of ICA^4,38,39^. Thus, the management of obstructive lesions identified on CCTA remains challenging^40^, resulting in low positive predictive value. In the cardiac catheterization lab, the functional significance of a stenotic lesion in the coronary arteries has been evaluated using instantaneous wave free ratio (iFR) and or FFR. The FFR technique requires induction of coronary hyperemia with adenosine, but the iFR technique does not need induction of coronary hyperemia^41^. These tests have been well validated and the new hybrid algorithm using iFR and FFR has been adapted in several catheterization laboratories across the United States^41^. Depending on the functional significance of the lesion, immediate revascularization of the stenotic lesion or deferred revascularization with medical treatment strategies could be adopted^13,42,43^. However, these techniques require an invasive approach and the peri-procedural complications associated with such an approach are not trivial. Also, ICA could result in unnecessary hospitalization and additional costs to the patient.

The ideal alternative would therefore be a non-imaging test that could assess the functional significance of a coronary stenotic lesion. The CTFFR was therefore proposed as a test to determine the functional significance of coronary lesions by use of computational fluid dynamics to the CCTA images^15,16^. Using multiplanar imaging and computer software, virtual hyperemia and computation modeling could be performed to estimate the functional significance of the lesions using CTFFR approach^17^. Calculation of CTFFR involves three main elements: physiological modeling of blood flow along with anatomical modeling of coronary arteries, and solution of governing equations of blood flow using numerical methods^16^. Since CCTA images are obtained during resting conditions, reducing resting microcirculatory resistance in the computational model simulates hyperemic response leading to computation of CTFFR^17^. As diseased and healthy vessels adapt to the amount of blood flow they carry, microcirculatory resistance is modeled. The luminal area of the feeding coronary artery is inversely proportional to the resistance of the vessel^17,44,45^. CTFFR therefore offers several advantages in terms of obviating the need for vasodilator administration, altering of the CCTA protocols or the need for additional radiation beyond what is normally expected during acquiring of CCTA images^46^.

In our meta-analysis, we observed that the CTFFR performed very well compared to the FFR in diagnosing functional significance of coronary stenosis and more importantly affords a very high negative predictive value to the test as seen on the Bayes nomogram. However, the studies did not report outcomes data and therefore at this time it is unclear if CTFFR strategy could result in improved prognosis by deferring coronary angiography and potentially revascularization. But it is clear from the results of our analysis that CTFFR offers more information than the traditional CCTA alone.

More recently, post hoc analysis based on hypothetical simulations of outcomes to assess clinicoeconomical effectiveness of using CTFFR to guide clinical decision making were reported^47,48^. Hlatky *et al*., projected that the patient stratified by CTFFR to undergo ICA and PCI had 30% lower costs and 12% fewer events at 1 year compared to the patients undergoing ICA with visually guided PCI^47^. In the other study by Kimura *et al*., a projected cost savings of 32% and 19% fewer adverse cardiac events were observed using a similar strategy as Hlatky *et al*.^48^. Further, Kim *et al*., demonstrated the improvement in diagnostic accuracy of CTFFR in identifying ischemic lesions by virtual coronary stenting using advanced computational models^20^. In the Prospective Longitudinal Trial of FFRCT: Outcomes and Resource Impacts (PLATFORM) study, the patients in the CTFFR arm had 32% lower costs of utilization but similar QOL scores in comparison to patients in ICA arm at 90 days^49^.

There are limitations to both the CTFFR and FFR techniques. In the CTFFR technique, artifacts induced from patient motion, blooming artifact from coronary calcification and insignificant contrast enhancement may limit the validity of the results^50^. In the case of FFR, procedural technique, the dose of vasodilator agent used to induce maximal hyperemia, distal microvascular resistance of the coronary arteries and high left ventricular end diastolic pressures can affect the results. Therefore, adequate attention to the patient’s clinical presentation and symptoms should also be factored in prior to proceeding with revascularization in addition to the above techniques.

Our study has several limitations. The studies included were few and had a small number of patients which decreases the robustness of the findings. Since, this is a meta-analysis all the weaknesses inherent to the individual studies will be inherited to our study. The included studies were published over a period of 5 years and there could have been alterations to the protocols in the study as well as improvements to the imaging and software algorithms that could contribute to the heterogeneity among the studies. Further, there could be inter-observed and intra-observer variability in interpreting the findings on these respective tests. Also, the dose of adenosine infused to induce coronary hyperemia could be variable among different centers across which these studies were performed contributing to varying findings among the studies. All the studies excluded patients with bypass grafts and therefore our results are not applicable to these patients. Lastly, publication bias although was tested and excluded could contribute to the findings of our study.

## Materials and Methods

### Data sources and searches

An extensive electronic search of Cochrane, SCOPUS and PubMed databases was performed for relevant articles using the following search terms: “computed tomography”, “CT angiography”, “computed tomography angiography”, “fractional flow reserve”, “FFR”, “fractional flow reserve, myocardial”. The search was restricted to publications in English and the final search was performed on April 2017.

### Study Selection

The following inclusion criteria were applied: (1) Age of the study population >18 yrs.; (2) CTFFR being the index test; (3) Design of the study being a diagnostic accuracy study; (4) FFR was chosen as the reference standard; (5) Data must allow construction of two-by-two contingency table. In case of multiple studies published from the same institution, the study reporting the highest number of subjects was included and the remaining studies were excluded from the final analysis.

### Data extraction and quality assessment

The initial search was performed independently by two reviewers and studies were selected for inclusion by mutual consensus. In case of disagreement, a third reviewer resolved disagreements between reviewers through discussion to achieve a consensus. Once the studies were selected for inclusion, the data was screened to meet the inclusion criteria and thereafter the following data points were extracted: true positive (TP), true negative (TN), false positive (FP) and false negative (FN). Additionally, cumulative demographic variables of patients included in the study were also extracted. We then performed meta-analysis at both the patient and the individual vessel levels. The quality of included studies was assessed using QUADAS-2 tool^51^.

### Data synthesis and Analysis

Continuous variables are presented as mean values and categorical data as percentages. The analysis of diagnostic performance of CTFFR was performed at both patient and individual vessel levels. We estimated the pooled sensitivity and specificity. In addition, negative predictive value (NPV), positive predictive value (PPV), LR+, LR− and DOR with respective 95% confidence intervals were performed. A weighted average of the pooled data on per-patient or per-vessel level was performed using bivariate random effects model^52^. The summary estimates with their 95% confidence intervals (CI) were calculated after anti-logit transformation of the mean logit sensitivity and specificity and respective standard errors. Statistical heterogeneity was defined as I^2^ statistic value greater than 50%^53^. The summary receiver operating characteristic curve (SROC) was derived using the logit estimates of sensitivity, specificity and their respective variances. This was then used to construct a hierarchical SROC curve and the area under the curve (AUC) estimated. An AUC range of 0.75–0.92 was considered to have good degree of diagnostic accuracy and an AUC range of 0.93–0.96 was considered to be a very good diagnostic test^54^. Publication bias was visually assessed using Deek’s funnel plot. The Deek’s funnel plot utilizes regression of diagnostic log odd’s ratio against 1/square root (effective sample size) and further weighting by effective sample size. A p-value of the slope coefficient of <0.10 indicated asymmetry and thereby publication bias^55^.

Meta-regression analysis was performed to identify potential sources of heterogeneity. We performed meta-regression for covariates including study design, sample size (less or more than 100), year of publication, region of study, quality of study, method of CTFFR appraisal, prevalence of coronary artery disease, proportion of hypertensive patients, proportion of diabetic patients, proportion of smokers and proportion of patients with dyslipidemia to identify potential sources of heterogeneity. Further, post-estimation models using Bayes methods were performed to compute the post-test likelihood of presence or absence of disease using a pre-test likelihood of having disease of 25%. This meta-analysis was performed in compliance with the Meta-analysis of Observational Studies in Epidemiology and the quality of reporting for meta-analysis and Preferred Reporting Items for Systematic reviews and Meta-Analyses (PRISMA) guidelines^56,57^. All the above analyses were performed in R, version 3.4.3, using “mada” package. The calculations for AUC and its confidence interval were performed using MetaDiSc version 1.4.

## Conclusion

The results of our study suggest that CTFFR has a good diagnostic accuracy and performs well in comparison to the FFR in the assessment of functional significance of the coronary stenotic lesions. The CTFFR technique is still evolving and needs further evaluation with assessment of clinical outcomes prior to widespread clinical adaptation.

## Electronic supplementary material

Supplementary Material
