# Supplementary material for: Comparison of Computed Tomography derived Fractional Flow Reserve to invasive Fractional Flow Reserve in Diagnosis of Functional Coronary Stenosis: A Meta-Analysis
Source: Sci Rep. 2018 Aug 1;8:11535. doi: 10.1038/s41598-018-29910-9 (PMC6070545; doi:10.1038/s41598-018-29910-9)

Supplementary material

Comparison of Computed Tomography derived Fractional Flow Reserve to invasive Fractional Flow Reserve in Diagnosis of Functional Coronary Stenosis: A Meta-Analysis.

Pradyumna Agasthi, MD[1],

Arun Kanmanthareddy, MD[2],

Charl Khalil, MD[1]

Obiora Egbuche, MD[3],

Vivek Yarlagadda, MD[4],

Rajesh Sachdeva, MD[3].

Reza Arsanjani, MD[1]

Table S1: Baseline Characteristics

Author	Year	Number of Patients	Age (Yrs) (Mean ± SD)		Male %	Smoking %	Diabetes %	Hypertension %	Dyslipidemia %
			Mean	SD					
Koo[14]	2011	103	62.7	9	72	36	26	65	65
Min[18]	2012	252	62.9	9	71	17.5	21.2	71.2	79.8
Norgaard[19]	2014	254	64	10	64	18	23	69	79
Kim[20]	2014	44	65	9	80		29	81	63
Renker[21]	2014	53	61.2	12	64	14	32	54	54
Coenen[22]	2015	106	61.4	9	77	25	19	59	63
De Geer[23]	2015	21	60	40-74	52.4	57.14	14.28		
Kruk[24]	2016	90	63.4	8	32	20.2	14.4	87.8	90
Zhang[25]	2016	21	52	10	76	10	10	52	62
Gaur[26]	2017	60	61	10	83	65	10	35	28
Kawaji[27]	2017	43	70.8	8	65	10	35	54	73
Ko[28]	2017	30	60	9	70	60	30	73.3	80
Kurata[29]	2017	21	69.6	9	76	43	33	71	38
Osawa[30]	2017	20	73	8	80	15	80	80	50
Packard[31]	2017	75	66	10	75	47	11	55	79
Shi[32]	2017	29	68.1	8	55.2	44.8	41.4	72.4	65.5
Yang[33]	2017	72	62.7	9	89	-	-	-	-

Table S2: Individual study estimates of diagnostic accuracy of FFRCT for detection of ischemia-causing lesions on a per-patient basis

Author	Year	TP	FP	FN	TN	Sensitivity %	95% CI	Specificity %	95% CI
Koo[14]	2011	50	9	4	40	92.59	82.11 - 97.94	81.63	67.98 – 91.24
Min[18]	2012	116	56	13	67	89.92	83.38 – 94.52	54.47	45.25 - 63.47
Norgaard[19]	2014	69	37	11	137	86.25	76.73 – 92.93	78.74	71.90 - 84.56
Kim[20]	2014	-	-	-	-	-	-	-	-
Renker[21]	2014	15	6	1	31	93.75	69.77 – 99.84	83.78	67.99 – 93.81
Coenen[22]	2015	-	-	-	-	-	-	-	-
De Geer[23]	2015	5	3	1	12	83.33	35.88 – 99.58	80	51.91 – 95.67
Kruk[24]	2016	31	14	10	35	75.61	59.70 – 87.64	71.43	56.74 – 83.42
Zhang[25]	2016	7	1	2	11	77	39.9 – 97.19	91.67	61.52 – 99.79
Gaur[26]	2017	-	-	-	-	-	-	-	-
Kawaji[27]	2017	-	-	-	-	-	-	-	-
Ko[28]	2017	-	-	-	-	-	-	-	-
Kurata[29]	2017	-	-	-	-	-	-	-	-
Osawa[30]	2017	-	-	-	-	-	-	-	-
Packard[31]	2017	-	-	-	-	-	-	-	-
Shi[32]	2017	15	5	1	8	93.5	69.77 – 98.89	61.54	35.58 – 86.14
Yang[33]	2017	-	-	-	-	-	-	-	-

Table S3: Individual study estimates of diagnostic accuracy of FFRCT for detection of ischemia-causing lesions on a per-vessel basis

Author	TP	FP	FN	TN	Sensitivity %	95% CI	Specificity %	95% CI
Koo[14]	51	18	7	83	87.93	76.7 – 95.0	82.18	73.30 – 89.08
Min[18]	121	96	30	160	80.13	72.87 – 86.18	62.5	56.26 - 68.45
Norgaard[19]	84	51	16	333	84	75.32 – 90.57	86.72	82.91 - 89.95
Kim[20]	29	6	5	8	85.29	68.94 – 95.05	57.14	28.86 – 82.34
Renker[21]	17	7	3	40	85	62.11 – 96.79	85.11	71.69 – 93.80
Coenen[22]	70	38	10	71	87.5	78.21 – 93.84	65.14	55.42 – 74.01
De Geer[23]	5	4	1	13	83.33	35.88 – 99.58	76.47	50.10– 93.19
Kruk[24]	31	15	10	40	75.61	59.70 – 87.64	72.73	59.04 –83.86
Zhang[25]	8	1	2	21	80	44.39 – 97.48	95.45	77.16 – 99.88
Gaur[26]	35	28	7	54	83.33	68.64 – 93.03	65.85	54.55 – 75.97
Kawaji[27]	26	20	2	22	92.86	76.50 – 99.12	52.338	36.42 – 68
Ko[28]	14	5	4	33	77.78	52.36 – 93.59	86.84	71.91 – 95.59
Kurata[29]	14	2	0	13	100	76.84 – 100	86.67	69.54 – 98.34
Osawa[30]	10	5	0	11	100	69.15 – 100	68.75	41.34 – 88.98
Packard[31]	66	18	3	120	95.65	87.82 – 99	86.96	80.17 – 92.08
Shi[32]	16	6	1	13	94.12	71.31 – 99.85	68.42	43.35 – 87.42
Yang[33]	47	19	7	65	87	75.1 – 94.6	77.38	66.95 – 85.8

Table S4: Additional covariates used for meta-regression analysis.

Author	Study design	Sample Size	Year	Location of publication	Quality	CT-FFR appraisal	Prevalence of Coronary Artery Disease (%)
Koo[14]	Multicenter prospective	>100	2011	Asia, America	High	FFR-CT core laboratory (HeartFlow, Inc. Redwood City, California, United States)	36.5
Min[18]	Multicenter prospective	>100	2012	Asia, America, Europe	High	FFR-CT core laboratory (HeartFlow, Inc. Redwood City, California, United States)	54.4
Norgaard[19]	Multicenter prospective	>100	2014	Asia,Europe,Australia	High	FFR-CT core laboratory (HeartFlow, Inc. Redwood City, California, United States)	32
Kim[20]	Multicenter retrospective	<100	2014	Asia, America, Europe	High	FFR-CT core laboratory (HeartFlow, Inc. Redwood City, California, United States)	100
Renker[21]	Single-center retrospective	<100	2014	America	High	Siemens cFFR, version 1.4; (Siemens Healthcare Forchheim, Germany)	30
Coenen[22]	Single-center retrospective	>100	2015	Europe	High	Siemens cFFR, version 1.4; (Siemens Healthcare Forchheim, Germany)	42
De Geer[23]	Single-center retrospective	<100	2015	Europe	Moderate	Siemens cFFR, version 1.4; (Siemens Healthcare Forchheim, Germany)	28.6
Kruk[24]	Single-center Prospective	<100	2016	Europe	High	Siemens cFFR, version 1.4; (Siemens Healthcare Forchheim, Germany)	42.7

Zhang[25]	Single-center Retrospective	<100	2016	Asia	High	ANSYS FLUENT (Canonsburg, Pennsylvania, United States	47.6
Gaur[26]	Single-center Prospective	<100	2017	Europe	Moderate	FFR-CT core laboratory (HeartFlow, Inc. Redwood City, California, United States)	58%
Kawaji[27]	Single-center Prospective	<100	2017	Asia	High	FFR-CT core laboratory (HeartFlow, Inc. Redwood City, California, United States)	49%
Ko[28]	Single-center Prospective	<100	2017	Australia	Moderate	CTFFR software (Toshiba Medical Systems Corporation, Otawara, Japan)	46.7
Kurata[29]	Single-center Retrospective	<100	2017	Asia	Moderate	Siemens cFFR, version 1.4; (Siemens Healthcare Forchheim, Germany)	48.3
Osawa[30]	Single-center Prospective	<100	2017	Asia	Moderate	FFR-CT core laboratory (HeartFlow, Inc. Redwood City, California, United States)	45
Packard[31]	Single-center Retrospective	<100	2017	America	Moderate	FFR-CT core laboratory (HeartFlow, Inc. Redwood City, California, United States)	33
Shi[32]	Single-center Retrospective	<100	2017	Asia	High	COMSOL Multiphysics software (COMSOL AB, Stockholm, Sweden),	47.2
Yang[33]	Single-center Prospective	<100	2017	Asia	Moderate	Siemens cFFR, version 1.4; (Siemens Healthcare Forchheim, Germany)	39

Table S5: Results of Meta-regression analysis

Meta-regression analysis per-patient			
Covariates	Meta-regression coefficient	95% CI	p-value
Study Design	1.567	(-1.819, 4.953)	0.3644
Sample size	-0.527	(-2.555, 1.501)	0.8358
Year of publication	-0.196	(-0.843, 0.451)	0.5527
Region (Asia vs Others)	0.826	(-1.643, 3.295)	0.5120
Quality	1.028	(-2.088, 4.145)	0.5179
CT-FFR appraisal (Siemens cFFR vs Others)	-0.236	(-2.470, 1.997)	0.5100
Prevalence of CAD	0.048	(-0.019, 0.114)	0.1597
Hypertension	0.025	(-0.146, 0.195)	0.7791
Diabetes	-0.119	(-0.245, 0.006)	0.0630
Smokers	0.059	(-0.052, 0.171)	0.2966
Dyslipidemia	-0.039	(-0.234, 0.135)	0.6581
Meta-regression analysis per-vessel			
Covariates	Meta-regression coefficient	95% CI	p-value
Study Design	0.402	(-2.611, 3.415)	0.7937
Sample size	-0.251	(-2.162, 1.661)	0.7971
Year of publication	-0.016	(-0.505, 0.473)	0.9491
Region (Asia vs Others)	-0.289	(-1.446, 0.869)	0.6250
Quality	0.606	(-0.553, 1.764)	0.3053
CT-FFR appraisal (Siemens cFFR vs Others)	0.188	(-1.045, 1.242)	0.7664
Prevalence of CAD	0.026	(-0.004, 0.056)	0.0843
Hypertension	0.047	(-0.005, 0.099)	0.0770
Diabetes	-0.028	(-0.069, 0.013)	0.1860
Smokers	-0.001	(-0.028, 0.026)	0.9291
Dyslipidemia	-0.026	(-0.069, 0.017)	0.2280

Figure S1. QUADAS-2 Results

Study	Risk of Bias				Applicability Concerns		
	Patient Selection	Index Test	Reference Standard	Flow and Timing	Patient Selection	Index Test	Reference Standard
Koo	☺	☺	☺	☺	☺	☺	☺
Min	☺	☺	☺	☺	☺	☺	☺
Norgaard	☺	☺	☺	?	☺	☺	☺
Kim	☺	☺	☺	?	☺	☺	☺
Renker	☺	☺	☺	?	☺	☺	☺
Coenen	☺	☺	☺	☺	☺	☺	☺
De Geer	☹	☺	☺	?	?	☺	☺
Kruk	☺	☺	☺	?	☺	☺	☺
Zhang	☺	☺	☺	?	☺	☺	☺
Gaur	☺	☺	☺	☺	☹	☺	☺
Kawaji	☺	☺	☺	?	☺	☺	☺
Ko	☹	☺	☺	?	☹	☺	☺
Kurata	☺	☺	☺	☺	☹	☺	☺
Osawa	☹	☺	☺	☺	☹	☺	☺
Packard	☹	☺	☺	☹	☹	☺	☺
Shi	☺	☺	☺	☺	☺	☺	☺
Yang	☺	☺	☺	?	☺	☺	☺

☺ Low Risk ☹ High Risk ? Unclear Risk

Figure S2 : QUADAS 2 DOMAIN- Graph for Bias

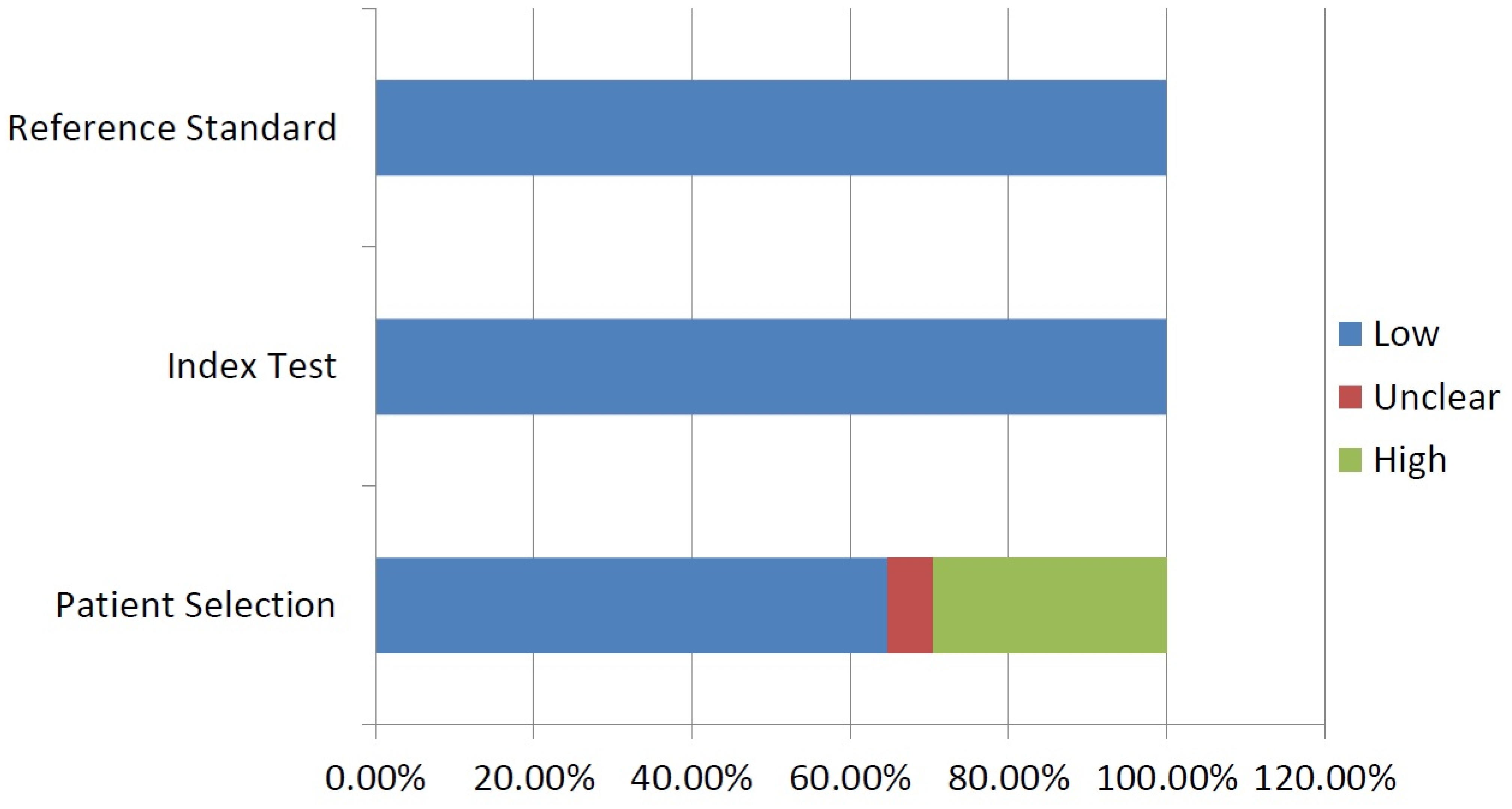

Figure S3：QUADAS 2 DOMAIN- Graph for Applicability

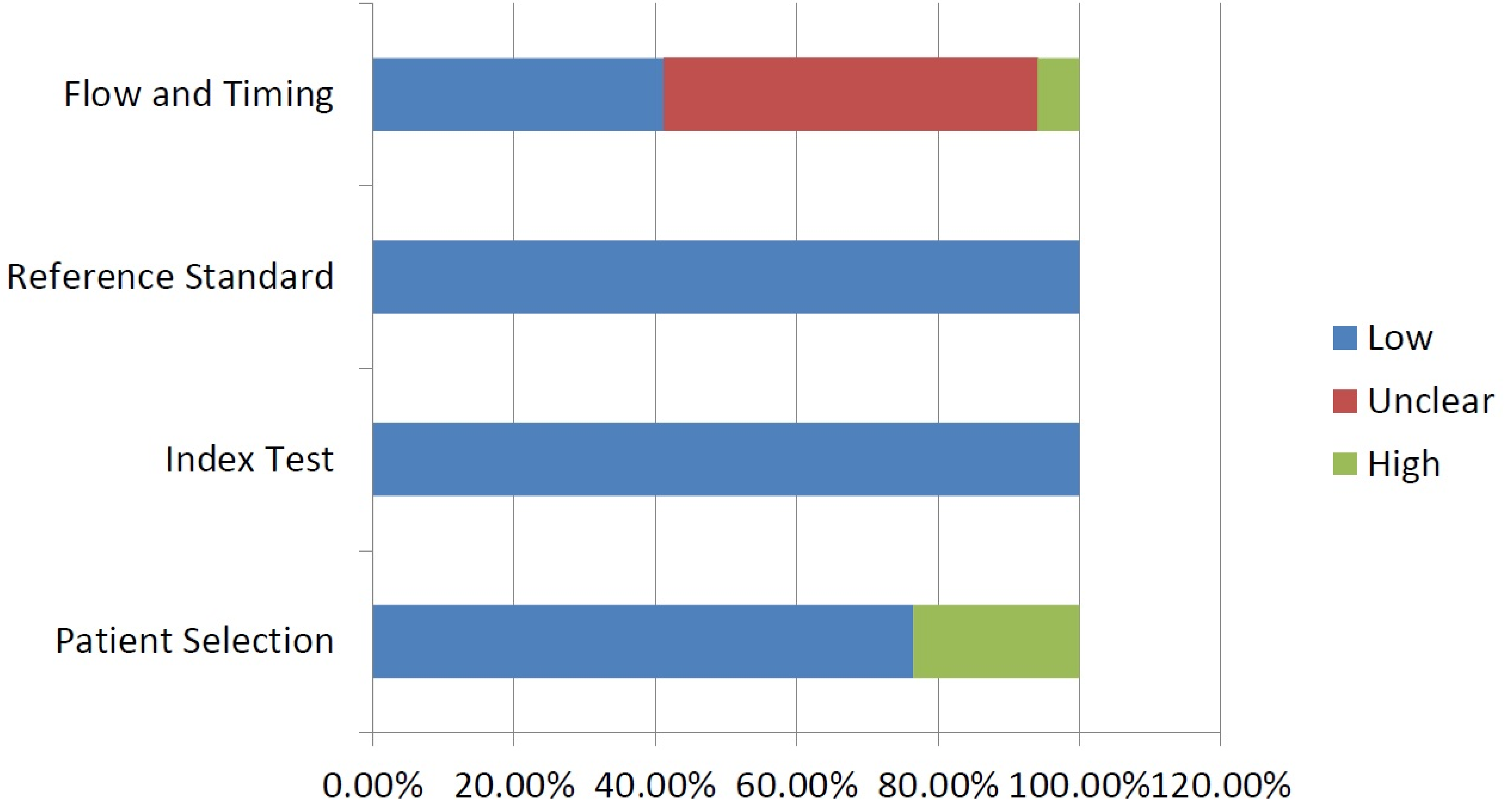

Figure S4: Patient Level Bayes Nomogram

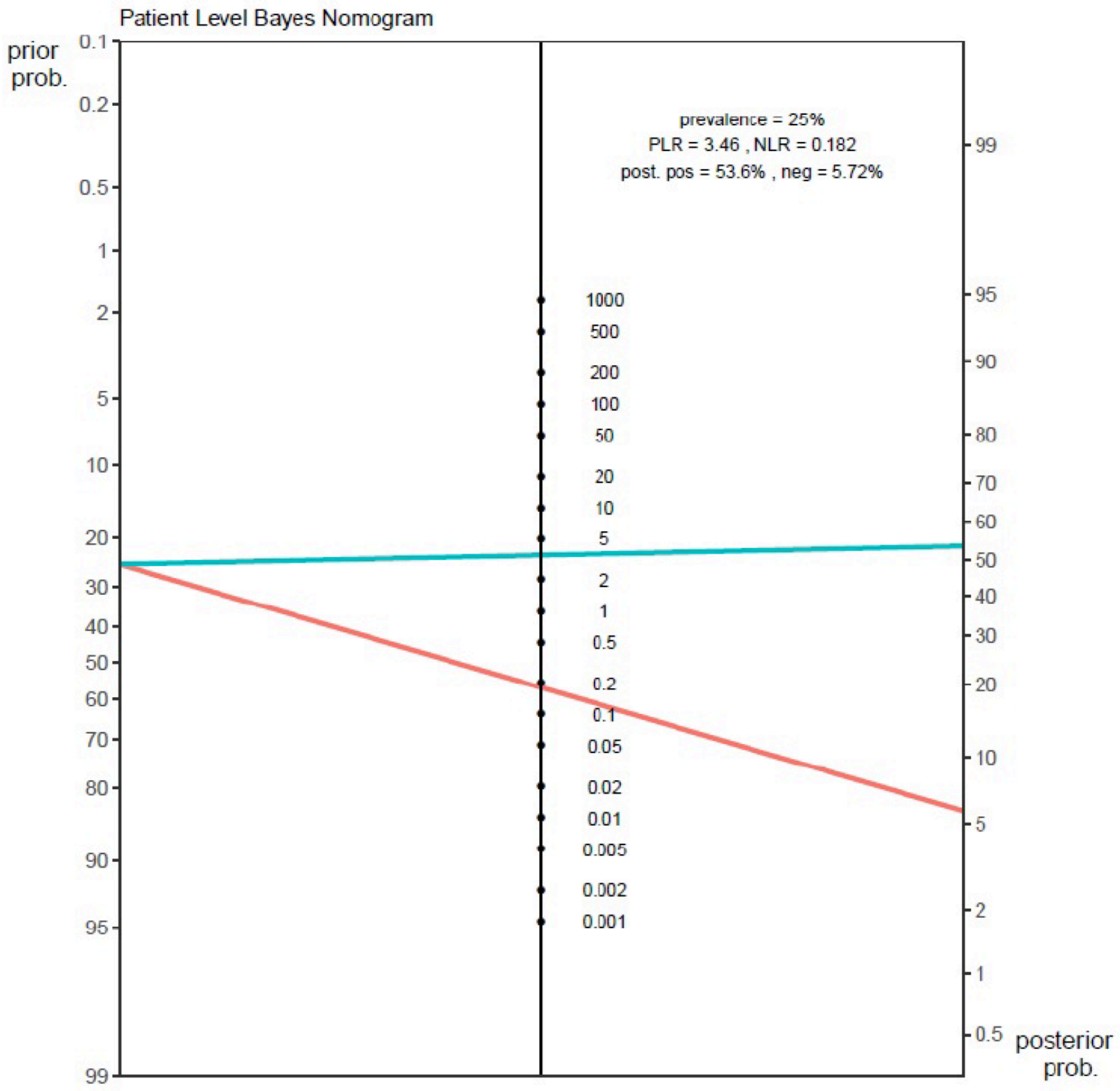

Figure S5: Individual Vessel Level Bayes Nomogram

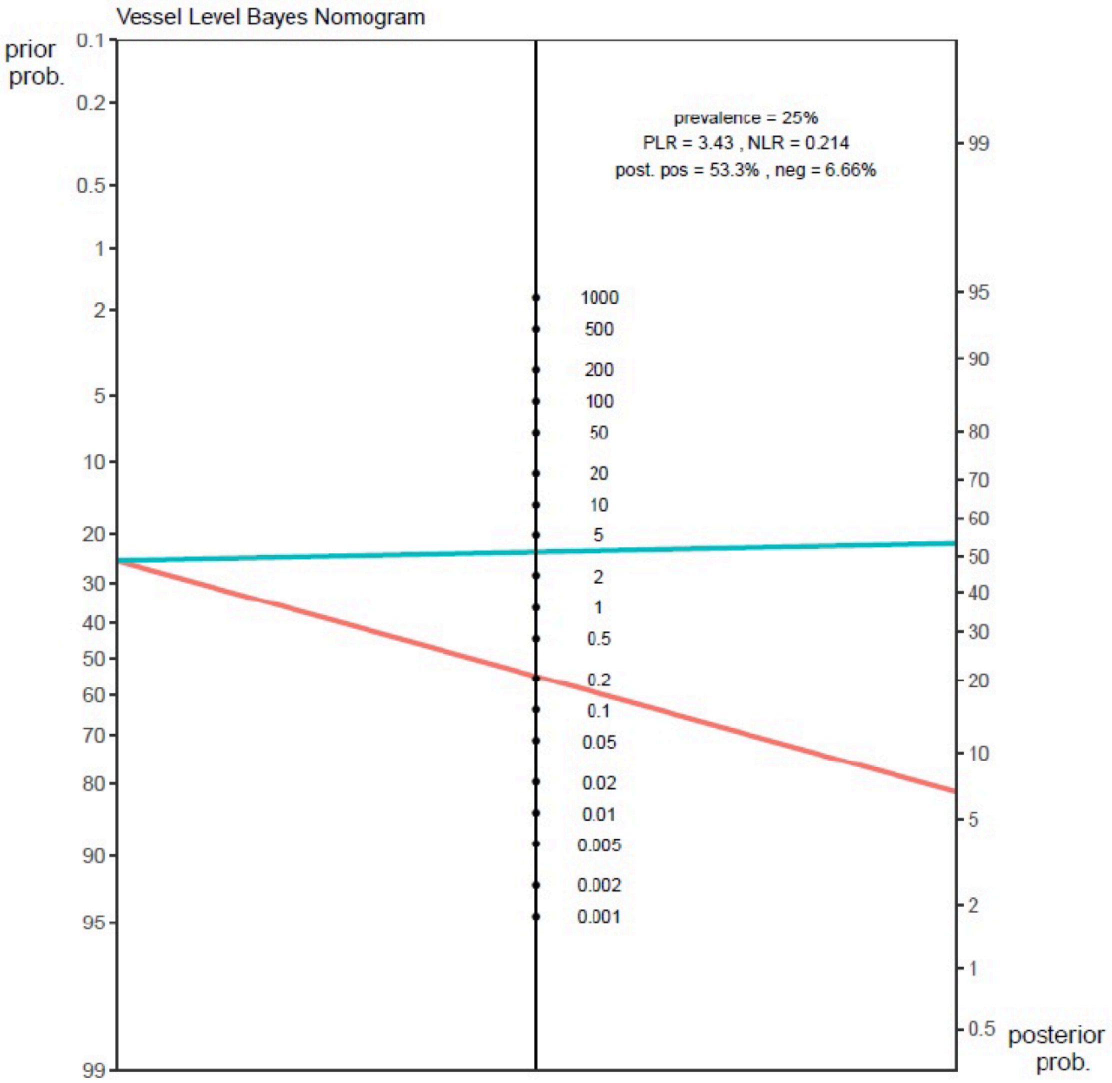

Supplement: Supplementary file 1 — Supplementary Material [file 41598_2018_29910_MOESM1_ESM.pdf]
